# Executive functions and adaptive behaviour in individuals with Down syndrome

**DOI:** 10.1111/jir.12897

**Published:** 2021-11-09

**Authors:** S. Onnivello, S. Colaianni, F. Pulina, C. Locatelli, C. Marcolin, G. Ramacieri, F. Antonaros, B. Vione, A. Piovesan, S. Lanfranchi

**Affiliations:** ^1^ Department of Developmental Psychology and Socialisation University of Padova Padova Italy; ^2^ Neonatology Unit St. Orsola‐Malpighi Polyclinic Bologna Italy; ^3^ Department of Experimental, Diagnostic and Specialty Medicine (DIMES), Unit of Histology, Embryology and Applied Biology University of Bologna Bologna Italy

**Keywords:** adaptive behaviour, behavioural phenotypes, BRIEF, Down syndrome, executive function, Vineland

## Abstract

**Background:**

Previous research has explored executive functions (EFs) and adaptive behaviour in children and adolescents with Down syndrome (DS), but there is a paucity of research on the relationship between the two in this population. This study aims to shed light on the profile of EFs and adaptive behaviour in DS, exploring the differences by age and investigating the relationship between these two domains.

**Method:**

Parents/caregivers of 100 individuals with DS from 3 to 16 years old participated in the study. The sample was divided into preschoolers (3–6.11 years old) and school‐age children (7–16 years old). Parents/caregivers completed either the Preschool Version of the Behaviour Rating Inventory of Executive Function (for children 2–6.11 years old) or the Second Edition of the same Inventory (for individuals 7 + years old). Adaptive behaviour was assessed with the Vineland Adaptive Behaviour Scale – Interview, Second Edition.

**Results:**

Findings suggest that individuals with DS have overall difficulties, but also patterns of strength and weakness in their EFs and adaptive behaviour. The preschool‐age and school‐age children's EF profiles differed slightly. While both age groups showed *Emotional Control* as a relative strength and *Working Memory* as a weakness, the school‐age group revealed further weaknesses in *Shift* and *Plan/Organise*. As concerns adaptive behaviour, the profiles were similar in the two age groups, with *Socialisation* as a strength, and *Communication* and *Daily Living Skills* as weaknesses, but with a tendency for preschoolers to obtain intermediate scores for the latter. When the relationship between EFs and adaptive behaviour was explored, *Working Memory* predicted *Communication* in the younger group, while in the older group the predictors varied, depending on the adaptive domains: *Working Memory* was a predictor of *Communication*, *Inhibit* of *Daily Living Skills*, and *Inhibit* and *Shift* of *Socialisation*.

**Conclusion:**

As well as elucidating the EF profiles and adaptive behaviour in individuals with DS by age, this study points to the role of EFs in adaptive functioning, providing important information for targeted interventions.

## Background

Down syndrome (DS) is the most common genetic cause of intellectual disability. In the majority of cases, it is caused by an extra chromosome 21 (trisomy 21) (Strippoli *et al*. 2019). Its estimated incidence is about one per 1000–1100 population (World Health Organisation, Genomic Resource Centre 2015). Although DS is associated with general developmental delays, specific vulnerabilities have been identified in various aspects of cognition (Chapman and Hesketh 2000; Lanfranchi *et al*. 2010). Difficulties with executive functions (EFs) have been demonstrated (Lanfranchi *et al*. 2010; Daunhauer, Fidler, Hahn *et al*. 2014; Daunhauer *et al*. 2017) and found associated with adaptation in academic, home and community settings in school‐aged children (Daunhauer, Fidler and Will 2014; Daunhauer *et al*. 2017), and with adaptive behaviour and employment in adulthood (Tomaszewski *et al*. 2018).

### Executive functions

The umbrella term EFs describes a set of higher order cognitive processes that are important for completing goals (Stuss and Benson 1986; Welsh *et al*. 1991; Zelazo *et al*. 1997). Several abilities have been classified as EFs, including working memory (i.e. the ability to keep information in mind and mentally work on it), shifting (i.e. the ability to transition from one task to another), planning and organisation (i.e. the ability to identify and select the steps required to obtain a goal), cognitive flexibility (i.e. the ability to switch from one cognitive framework to another), monitoring (i.e. the ability to check, update and keep track of information about more than one task and to recognise when the next step of a task or a switch to another task is required) and emotional control (i.e. the ability to experience, express and modulate emotional experiences) (Pennington and Ozonoff 1996; Miyake *et al*. 2000; Friedman *et al*. 2006). These functions are thought to be related, but distinct, as suggested by low correlations between various EF tasks (Miyake *et al*. 2000). Several studies have conducted laboratory tests to analyse EFs in individuals with DS (e.g. Kogan *et al*. 2009; Lanfranchi *et al*. 2010; Daunhauer and Fidler 2013). The findings suggest impairments with respect to participants' mental age in various EFs, such as verbal and visuospatial working memory, the verbal component of inhibition, shifting and planning skills, and sustained attention (e.g. Lanfranchi *et al*. 2010; Borella *et al*. 2013; Carney *et al*. 2013; Costanzo *et al*. 2013; Esbensen *et al*. 2019). More recently, a growing number of studies have focused on assessing EFs in daily living situations. A measure widely used in this context is the Behaviour Rating Inventory of Executive Function (BRIEF; Gioia *et al*. 2000) or its version for preschoolers, BRIEF‐P (Gioia *et al*. 2003). These rating scales are completed by parents and/or teachers to assess EF‐related behaviour at school and at home. The BRIEF‐P was conceived for use with children aged between 2 and 5 years. Items are grouped into five subscales, assessing *Inhibition*, *Shifting*, *Emotional Regulation*, *Working Memory* and *Planning/Organisation*. Although it is intended for preschool‐age children, several studies have also applied it to older children with DS (using it outside the normative range), judging the items more appropriate for their mental age (Lee *et al*. 2011; Daunhauer, Fidler, Hahn *et al*. 2014; Pritchard *et al*. 2015). These studies demonstrated that, when used with this population, the BRIEF‐P is reliable, stable and sensitive to age, and it detects a profile of impairment consistent with the one found in studies using laboratory measures (Lee *et al*. 2011; Liogier d'Ardhuy *et al*. 2015). The BRIEF is the version for older children (6 to 18 years old) developed to explore types of behaviour more typical of school‐age children. It includes three additional clinical subscales: *Self‐Monitoring*, *Initiation* and *Task Monitoring*. Previous studies using the BRIEF for older children and adolescents with DS showed that it retained its psychometric properties and appropriateness when used with this population (e.g. Esbensen *et al*. 2019). Proxy‐report measures (like the BRIEF and BRIEF‐P) have the advantage of ecological validity and may serve as a more accurate measure of ‘successful EF goal pursuit’ in everyday life than laboratory‐based, direct assessments (Toplak *et al*. 2013). Indirect measures of EFs also seem to be particularly appropriate for individuals with DS, avoiding methodological issues frequently encountered with laboratory tests, such as problems with understanding instructions or floor effects (Pulina *et al*. 2019). Previous studies using the BRIEF and BRIEF‐P found an overall impairment in EFs in individuals with DS (Daunhauer, Fidler, Hahn *et al*. 2014; Lee *et al*. 2011, 2015; Loveall *et al*. 2017), but also a particular profile with relative strengths and weaknesses. Studies focusing on preschoolers found this age group relatively stronger in *Emotional Control* and *Shift* and weakest in *Working Memory* (Loveall *et al*. 2017). The strongest skills in school‐age children appear to be *Emotional Control* and *Organisation of Materials*, while the weakest concern *Working Memory*, *Monitor*, *Plan/Organise* and *Shift* (Daunhauer, Fidler, Hahn *et al*. 2014; Lee *et al*. 2011, 2015; Loveall *et al*. 2017). Not many studies have looked at age‐related differences in the EFs of individuals with DS (Lee *et al*. 2015; Loveall *et al*. 2017). In one cross‐sectional study, Lee *et al*. (2015) explored the BRIEF profile for EFs and the effects of age in a sample of individuals from 4 to 24 years old. They found that EF difficulties in individuals with DS remain much the same throughout childhood and into young adulthood (up to 24 years old), suggesting a stable BRIEF profile over time. On the other hand, a cross‐sectional study by Loveall *et al*. (2017) comparing preschoolers (2–5 years old) with school‐age children (6–18 years old) found this profile only partially stable. In both groups, they identified *Emotional Control* as a relative strength, *Working Memory* as a weakness and *Inhibit* somewhere in between. The picture changed in some aspects over time, however. *Shift* went from being a strength in preschoolers to a weakness in the school‐aged group. *Plan/Organise* was an intermediate‐level ability in the preschool group but became a weakness in the school‐age children.

### Adaptive behaviour

The term ‘adaptive behaviour’ refers to the conceptual, practical and social skills that individuals use in their everyday lives (Schalock *et al*. 2010). Conceptual skills involve both receptive and expressive language, reading, writing, math reasoning and understanding the concepts of time and money. Social skills involve awareness of others' thoughts and feelings, friendship skills, the ability to respect social rules, and social judgement. Practical skills involve personal care, job responsibilities, money management and work task organisation (Schalock *et al*. 2010). Difficulties with aspects of adaptive behaviour are a part of the definition of intellectual disability, making adaptive behaviour a crucial dimension to consider in individuals with DS (Schalock *et al*. 2010).

It is important to assess adaptive behaviour to see how individuals function, and take appropriate steps to improve their autonomy, and their quality of life as a consequence. Previous studies exploring adaptive behaviour in DS used the Vineland Adaptive Behaviour Scales, Second Edition (VABS‐II, Sparrow *et al*. 2005), which assess skills indirectly – usually by means of parental reports in the case of children. The skills considered include communication (e.g. understanding and expressing language), daily living skills (e.g. hygiene and household chores), socialisation (e.g. forming relationships and coping) and motor skills (e.g. going up and down the stairs or using scissors). When this tool has been used to assess children and adolescents with DS between 1 and 17 years old, it has identified a profile characterised by strengths in *Socialisation* and weaknesses in *Communication* and *Motor Skills* (Dykens *et al*. 2006; Will *et al*. 2018; Spiridigliozzi *et al*. 2019). A global impairment in adaptive behaviour emerges for infants and toddlers with DS (5–45 months old) by comparison with typically developing children. The former have difficulties across all domains already in the first year of life (Will *et al*. 2018), with their standard scores declining as they grow older (Will *et al*. 2018; Spiridigliozzi *et al*. 2019). Young children with DS also show a deceleration in adaptive trends as they grow up, the most pronounced discrepancies between DS and typical development involving motor and communication skills (Will *et al*. 2018). The profile remains fairly stable over time, although toddlers seem to present a more varied picture (with strengths in *Socialisation* and weaknesses in *Communication*), while 12‐year‐olds show a flatter profile (Van Duijn *et al*. 2010). The profile seems to persist through adolescence and young adulthood, before a decline in *Communication* occurs beyond the age of 22 (Spiridigliozzi *et al*. 2019).

It is well known that EFs are fundamental to planning, organising and monitoring everyday activities, and also for adaptive behaviour. Previous studies on typically developing individuals found a relationship between EFs and adaptive behaviour, demonstrating that the former contributes to the latter. For example, working memory, shifting and inhibition have been found to be related to communication (Mazuka *et al*. 2009; Kaushanskaya *et al*. 2017). Inhibitory control also relates significantly to adaptive behaviour in early childhood, and to socialisation in particular, helping an individual to avoid inadequate responses and to adjust to social norms (Diamond 2013; Benavides‐Nieto *et al*. 2017). A relation between EFs and adaptive behaviour has also been demonstrated in individuals with autism spectrum disorders (e.g. Gilotty *et al*. 2002; Gardiner and Iarocci 2018) or intellectual disabilities (Gligorović and Buha 2014). To our knowledge, only one such study is available on DS (Sabat *et al*. 2020), which focuses on adolescents aged 12–17 years. EFs (working memory, inhibition and cognitive flexibility) were assessed using laboratory tasks, and adaptive behaviour (conceptual, social and practical) was rated by parents and teachers. Working memory predicted conceptual adaptive behaviour rated by parents, while inhibition and flexibility predicted conceptual adaptive behaviour rated by teachers. This difference may stem from the fact that different settings (home vs. school) make different demands on the child. It is important to explore these relationships in more depth to clarify which skills are worth targeting in early intervention for children with DS to improve their adaptive behaviour – and their quality of life as a result.

### The present study

Given the above considerations and previous literature, the aim of the present study is to shed further light on the strengths and weaknesses in the EFs and adaptive behaviour of preschoolers and school‐age children with DS. The relationship between the two domains is also explored. In particular, the present study with DS poses the following research questions.

1. Do preschoolers and school‐age children with DS have the same relative strengths and weaknesses in EFs? In the light of the study by Loveall *et al*. (2017), both groups are expected to have lower scores than the normative group. Preschoolers are expected to have a profile characterised by strengths in *Emotional Control* and *Shift*, and a weakness in *Working Memory*, while school‐age children are expected to have a more complex profile with *Emotional Control* and *Organisation of Materials* as strengths, and *Working Memory*, *Monitor*, *Plan/Organise* and *Inhibit* as weaknesses.
2. Do preschoolers and school‐age children with DS have the same relative strengths and weaknesses in adaptive behaviour? Based on the findings of previous studies (e.g. Spiridigliozzi *et al*. 2019) and the definition of intellectual disability, lower scores than in the normative group can be expected in all the adaptive behaviour domains considered, with a more mixed profile for preschoolers, and with strengths in *Socialisation* and weaknesses in *Communication* for school‐age children.
3. Is the relationship between EFs and adaptive behaviour the same in preschoolers and school‐age children with DS? In line with previous studies on typically developing children (e.g. Mazuka *et al*. 2009; Diamond 2013; Benavides‐Nieto *et al*. 2017; Kaushanskaya *et al*. 2017), other populations (e.g. Gligorović and Buha 2014; Gardiner and Iarocci 2018) and adolescents with DS (Sabat *et al*. 2020), a relationship between EFs and adaptive behaviour can be expected. In addition, because adaptive behaviour requires more complex skills for school‐age children than at preschool age, there are expected to be more correlations in the older group than in the younger one.

Shedding more light on the EF profiles and adaptive behaviour of children/adolescents with DS, identifying any age‐related differences, and understanding the relationship between the two domains in DS can elucidate developmental patterns and support targeted interventions to improve long‐term outcomes.

## Method

### Participants

One hundred parents/caregivers of individuals with DS took part in the study, after giving their informed consent. They were recruited during the annual follow‐up of the individuals with DS at the Unit of Neonatology of St. Orsola‐Malpighi Polyclinic in Bologna, Italy. The sample was divided into two groups based on the children's age and education level: preschoolers (aged between 3 and 6.11 years) and school‐age children (between 7 and 16 years old). In the school system in Italy, as in the vast majority of European countries, children start school at 6 years of age. However, as all children with DS in our country are included in mainstream schools, it is not infrequent for parents to decide, by agreement with the child's clinician, to keep a child in kindergarten a year longer to give them more time to acquire the fundamental skills needed in first grade at primary school. That is why participants in our group of school‐age children were 7 or more years old. There were 40 children in the preschooler group and 60 in the school‐age group. Participants' characteristics are shown in Table 1. No differences emerged between the groups in terms of sex, race, or the mothers' or fathers' education.

**TABLE 1:** Participants' characteristics (*n* = 100)

	Preschoolers (*n* = 40)	School‐age children (*n* = 60)	Differences between groups
Sex (% male)	62.5	63.3	*X* ^2^ = 0.007, *P* = 0.93
Chronological age (months)	57.15 (15.12)	138.96 (28.91)	*t* = 15.39, *P* < 0.001
Race (% Caucasian)	95	100	*X* ^2^ < 0.001, *P* = 1.00
Mothers' education (% college degree or higher)	75	56.6	*X* ^2^ = 1.48, *P* = 0.22
Fathers' education (% college degree or higher)	50	50	*X* ^2^ = 1.19, *P* = 0.28

### Measures

#### Behaviour Rating Inventory of Executive Function – Preschool Version

The BRIEF‐P (Gioia *et al*. 2003) is a standardised rating scale designed to measure EFs in children 2–5.11 years old. It was completed by the parents or caregivers for the children with DS. The BRIEF‐P presents a series of 63 statements regarding a child's behaviour. For each statement, parents are asked to rate how often (never = 1, sometimes = 2, or often = 3) each type of behaviour has been a problem in the previous 6 months. Higher scores indicate more severe problems. The BRIEF‐P yields *T*‐scores (*M* = 50, *SD* = 10), which are standardised scores based on the age and sex of the individual being described. There are five scales: *Inhibit*, *Shift*, *Emotional Control*, *Working Memory* and *Plan/Organise*, which together give rise to three index scales: *Inhibitory Self‐Control* (*ISCI* = *Inhibit* + *Emotional Control*), *Flexibility* (*FI* = *Shift* + *Emotional Control*) and *Emergent Metacognition* (*EMI* = *Working Memory + Plan/Organise*). A composite score is obtained as well, called the *Global Executive Composite* score. The BRIEF‐P parent form has a good internal consistency (0.80–0.95) and a good test–retest reliability (0.78–0.90; Gioia *et al*. 2003).

Following a procedure already used in the field (e.g. Lee *et al*. 2011; Daunhauer, Fidler, Hahn *et al*. 2014; Pritchard *et al*. 2015), the BRIEF‐P was used in the present study for children from 3 to 6.11 years old (preschoolers), as the items in the BRIEF‐P are more appropriate than those in the BRIEF for children attending preschool. Raw scores from each of the scales and indexes were used to generate age‐referenced and sex‐referenced normative *T*‐scores. In this study, CA was used to generate age‐referenced *T*‐scores. However, considering that normative data are up to 5.11 years old, for children aged between 6 and 6.11 years, *T*‐scores were calculated referring to the normative data for the oldest age range, that is, 4–5.11 years.

#### Behaviour Rating Inventory of Executive Function – Second Edition

The BRIEF 2 (Gioia *et al*. 2000) is a standardised rating scale designed to measure EFs in individuals aged 6–18 years. The BRIEF 2 parent form consists of 86 items. The rating format and *T*‐score norms are the same as for the BRIEF‐P. The BRIEF 2 contains nine scales that partially overlap with those of the BRIEF‐P: *Inhibit*, *Self‐Monitor*, *Shift*, *Emotional Control*, *Initiate*, *Working Memory*, *Plan/Organise*, *Task‐Monitor* and *Organisation of Materials*. The scales are combined to calculate three indexes: *Behaviour Regulation* (*BRI* = *Inhibit* + *Self‐Monitor*), *Emotion Regulation* (*ERI* = *Shift* + *Emotional Control*) and *Cognitive Regulation* (*CRI = Initiate* + *Working Memory* + *Plan/Organise* + *Organisation of Materials* + *Task‐Monitor*). Finally, a *General Executive Composite* score is calculated from all the scales. The BRIEF 2 parent form has a good internal consistency (0.80–0.98) and a good test–retest reliability (0.72–0.88).

Both versions of the BRIEF have already been used successfully with parents of individuals with DS (e.g. Edgin *et al*. 2010; Loveall *et al*. 2017). There are also published studies showing correlations between the BRIEF scales and laboratory‐based assessments of EFs, minimal floor performance, and adequate test–retest reliability in DS (Edgin *et al*. 2010; Liogier d'Ardhuy *et al*. 2015). Esbensen *et al*. (2019) demonstrated, moreover, that the BRIEF and its subscales generally perform in a psychometrically sound manner when applied to children with DS.

#### Vineland Adaptive Behaviour Scales, Second Edition – Survey Interview Form

The VABS‐II – Survey Interview Form (Sparrow *et al*. 2005) is a semi‐structured interview for parents/caregivers of individuals aged from birth to 90 years. It investigates adaptive behaviour across four domains: *Communication*, *Daily Living Skills*, *Socialisation* and *Motor Skills* (for ages 0–6 years only). The *Communication* domain contains three subdomains assessing how well an individual understands language (*Receptive*), produces language (*Expressive*) and understands how to use letters and words, as well as how to read and write (*Written*). The *Daily Living Skills* domain contains three subdomains concerning an individual's skills in eating, dressing and hygiene (*Personal*), household tasks (*Domestic*), and time and money management, technology and job‐related skills (*Community*). The *Socialisation* domain contains three subdomains covering an individual's relationships (*Interpersonal Relationships*), recreational skills (*Play and Leisure*), and how an individual demonstrates sensitivity and responsibility (*Coping Skills*). The *Motor Skills* domain includes two subdomains concerning fine and gross motor skills. Items are scored on a 0–2 scale indicating the frequency with which an individual uses a given skill autonomously: usually (2), sometimes (1) or never (0). Raw scores are converted into standard scores (*M* = 100; *SD* = 15), and a composite standard score, the *Adaptive Behaviour Composite*, is obtained from the standard scores in the four domains.

High internal consistencies have been reported across all VABS‐II domains (*r*s = 0.70–0.95), and a high inter‐rater reliability has been reported for the Survey Interview Form (*r*s = 0.68–0.95).

### Procedure

The present data were collected as part of a broader project aiming to explore the correlation between genotype and phenotype in DS. All participants were admitted to the Unit of Neonatology of St. Orsola‐Malpighi Polyclinic in Bologna, Italy, and the study was proposed during routine annual follow‐up visits for children with DS. Written consent was obtained from the participating parents/caregivers before they were interviewed in a quiet room at the Department of Developmental Psychology and Socialization in Padova, Italy, by a psychologist who first administered the Vineland‐II, then participants completed the BRIEF‐P or BRIEF 2 questionnaire under the psychologist's supervision, and the psychologist was available to explain any items they found unclear.

### Analysis plan

Descriptive statistics, Student's *t*‐test, repeated‐measures ANOVAs and regression‐based curve estimates were used in the analyses.

First, to answer the question of whether preschoolers and school‐age children with DS have the same strengths and weaknesses in EFs, descriptive statistics were calculated on *T*‐scores, and the percentages of clinically elevated scores were recorded. Student's *t*‐tests were used to see how the children and adolescents with DS compared with the normative group to identify similarities and differences with respect to typical development. Repeated‐measures ANOVAs were run to describe the profile of strengths and weaknesses in EFs separately for each group (since the BRIEF‐P and BRIEF 2 scales partially differ), considering first the indices and then the scales. In this analysis, the Greenhouse–Geisser adjustment was applied to the *P* values (reported as *P*_gg_) when the assumption of sphericity was violated. Post‐hoc *t*‐tests were two‐tailed, and the *P* values were corrected for the analysis of multiple comparisons using the Bonferroni method (i.e. the alpha value was divided by the number of comparisons). Cohen's *d* was calculated to establish the magnitude of the effects, where the rule of thumb for effect sizes was as follows: *d* (0.01) = very small, *d* (0.2) = small, *d* (0.5) = medium, *d* (0.8) = large, *d* (1.2) = very large and *d* (2.0) = huge (Sawilowsky 2009). Only the five scales that the two tools have in common (*Inhibit*, *Shift*, *Emotional Control*, *Working Memory* and *Plan/Organise*) were considered when comparing the EF profiles of the preschoolers and school‐age children, using repeated‐measures ANOVAs.

As done for EF, for the question of whether preschoolers and school‐age children with DS have the same strengths and weaknesses in adaptive behaviour, descriptive statistics were calculated on standardised scores, along with the percentages of clinically elevated standardised scores for preschoolers and school‐age children.

To elucidate the strengths and weaknesses in adaptive behaviour in the two groups, and to compare the two profiles, a repeated‐measures ANOVA was run with Scale as the within factor and Group as the between factor.

Finally, to explore whether the relationship between EFs and adaptive behaviour is the same in preschoolers and school‐age children with DS, bivariate correlations were run separately for the two groups, to examine the relationship between the EF indexes (BRIEF‐P and BRIEF 2) and the children's adaptive behaviour (Vineland‐II). Regression models were used to explore the combined effect of the EFs considered on adaptive behaviour. For each model, age was entered first, followed by the indexes, and each Vineland‐II scale was the outcome variable. Following a procedure already used in the field (e.g. Esbensen *et al*. 2021), when an index was found associated with a Vineland‐II scale, further analyses were run to detect which scale of the index showed the strongest association.

## Results

### Executive functions

#### Executive functions in the preschooler group

Table 2 shows the descriptive statistics, the percentages of clinically elevated *T*‐scores (>65) and the results of Student's *t*‐test comparing the mean *T*‐score of the group with the norm of 50.

**TABLE 2:** Percentages of clinically high *T*‐scores, means, standard deviations (SDs) and one‐sample *t*‐test results for children 3–6.11 years old on the BRIEF‐P

	%CE^†^	Mean	SD	*t* ^‡^	*P* value	Cohen's *d*
Inhibit	20	55.25	11.98	2.77	0.009	0.43
Shift	10	52.08	11.98	1.13	0.27	0.18
Emotional Control	5	46.10	7.96	−3.09	0.004	0.49
Working Memory	47.5	65.73	10.55	9.43	<0.001	1.49
Plan/Organise	15	57.25	7.86	5.83	<0.001	0.92
Inhibitory Self‐Control Index	32.5	51.33	10.65	0.79	0.44	0.12
Flexibility Index	5	48.63	9.11	−0.95	0.35	0.51
Emergent Metacognition Index	40	63.45	9.56	8.90	<0.001	1.40
Global Executive Composite score	20	57.65	8.99	5.38	<0.001	0.85

^†^ Percentage of individuals with DS reportedly in the clinically high scoring range (*T* ≥ 65).

^‡^ Comparison with normative *T*‐score of 50.

Among the children with DS aged 3–6.11 years, almost half of the sample had clinically elevated T‐scores for *Working Memory* and the *Emergent Metacognition Index* (47.5% and 40%, respectively). In contrast, only 5% of the sample had clinically elevated *T*‐scores for *Emotional Control* and the *Flexibility Index*.

*T*‐scores obtained from the *Global Executive Composite* scores were significantly above the norm of 50. Of the three indexes, however, only the *Emergent Metacognition Index* was significantly above the norm, suggesting that the main EF deficit in DS at this age concerns *Emergent Metacognition. T*‐scores for the five scales were also compared with the norm, using a Bonferroni correction (*α* 0.05/5 = 0.01). Some subscales indicated significant difficulties, suggesting a profile of strengths and weaknesses, the former in *Emotional Control* and the latter in *Inhibit*, *Working Memory* and *Plan/Organise*.

**FIGURE 1:**
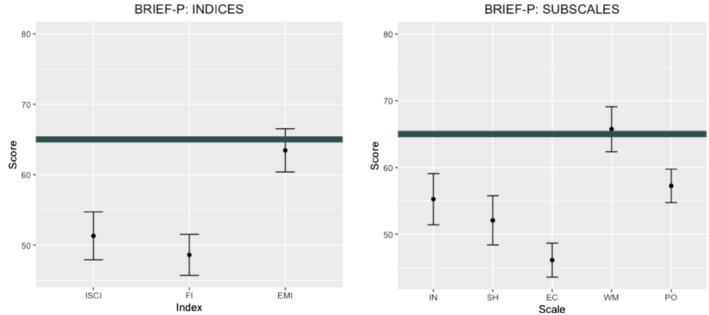
BRIEF‐P scores in the preschooler group. BRIEF‐P, Behaviour Rating Inventory of Executive Function – Preschool Version; EMI, Emergent Metacognition Index; FI, Flexibility Index; IN, Initiate; INH, Inhibit; ISCI, Inhibitory Self‐Control Index; PO, Plan/Organise; SH, Shift; WM, Working Memory. The thick line represents the BRIEF‐P cut‐off of 65. Scores higher than this value are in the clinically elevated range. [Colour figure can be viewed at wileyonlinelibrary.com]

Then, the EF profiles were investigated by running two different repeated‐measures ANOVAs. In one, the three indexes were dependent variables and *Index* was the within‐group variable. In the other, the seven scales were dependent variables, and *Scale* was the within‐group variable. *T*‐scores were considered for both analyses. A significant effect of *Index* emerged (*F*_2,78_ = 44.21, *P* < 0.001, *η*_p_^2^ = 0.53), and subsequent post‐hoc analyses showed higher scores in *Emergent Metacognition* than in *Inhibitory Self‐Control* (*Mdiff*. = 12.13, *P* < 0.001, *d* = 1.01) or *Flexibility* (*Mdiff*. = 14.83, *P* < 0.001, *d* = 1.46). There was also a significant effect of *Scale*, *F*_2.87,112.02_ = 29.70, *P*_gg_ < 0.001, *η*_p_^2^ = 0.43, and post‐hoc analyses identified significant differences between *Emotional Control*, which had the lowest score (and was therefore the greatest strength), and all the other scales: *Inhibit* (*Mdiff*. = −9.15, *P* < 0.001, *d* = 0.93), *Shift* (*Mdiff*. = −5.96, *P* = 0.014, *d* = 0.54), *Working Memory* (*Mdiff*. = −19.63, *P* < 0.001, *d* = 1.75) and *Plan/Organise* (*Mdiff*. = −11.15, *P* < 0.001, *d* = 1.18). *Working Memory*, which had the highest score (making it the greatest weakness), differed significantly from *Inhibit* (*Mdiff*. = 10.47, *P* < 0.001, *d* = 0.80), *Shift* (*Mdiff*. = 13.65, *P* < 0.001, *d* = 1.09) and *Plan/Organise* (*Mdiff*. = 8.47, *P* < 0.001, *d* = 1.05). Figure 1 shows the profiles, considering both the indexes and the scales.

#### Executive functions in the school‐age group

Table 3 shows the descriptive statistics, the percentages of clinically elevated *T*‐scores (>65) and the results of Student's *t*‐test comparing the mean *T*‐score of the group with the norm of 50.

**TABLE 3:** Percentages of clinically high *T*‐scores, means, standard deviations (SDs) and one‐sample *t*‐test results for children 7–16 years old on the BRIEF 2

	%CE^†^	Mean	SD	*t* ^‡^	*P* value	Cohen's *d*
Inhibit	15	54.95	11.77	3.25	0.002	0.42
Self‐Monitor	11.67	55.53	8.79	4.87	<0.001	0.63
Shift	36.67	60.73	13.38	6.21	<0.001	0.80
Emotional Control	10	48.43	10.37	−1.17	0.25	0.15
Initiate	26.67	59.00	10.17	6.86	<0.001	1.89
Working Memory	28.33	59.82	7.69	9.88	<0.001	1.28
Plan/Organise	25	59.78	8.13	9.32	<0.001	1.20
Task‐Monitor	31.67	60.42	10.81	7.46	<0.001	0.96
Organisation of Materials	11.67	51.75	9.53	1.42	0.160	0.18
Behaviour Regulation Index	16.67	55.43	9.40	4.48	<0.001	0.58
Emotion Regulation Index	16.67	54.52	10.97	3.19	0.002	0.41
Cognitive Regulation Index	25	59.58	7.22	10.28	<0.001	1.33
Global Executive Composite score	26.67	58.48	8.65	7.60	<0.001	0.98

† Percentage of individuals with DS reportedly in the clinically high scoring range (*T* ≥ 65).

^‡^ Comparison with normative *T*‐score of 50.

For children with DS aged 7–16 years, the highest percentages of clinically elevated scores emerged for *Shift* (37%) and *Task‐Monitor* (32%), and the lowest percentages being for *Emotional Control* (10%) and *Organisation of Materials* (12%).

*T*‐scores obtained from the *Global Executive Composite* scores were significantly above the norm of 50, and so were those for the three indexes concerning *Behaviour Regulation*, *Emotional Regulation* and *Cognitive Regulation*. The *T*‐scores for each of the nine scales were also compared with the norm of 50, using Bonferroni's correction (*α* 0.05/9 = 0.006). The scores were significantly higher than 50 for almost all the scales (*Inhibit*, *Self‐Monitor*, *Shift*, *Initiate*, *Working Memory*, *Plan/Organise* and *Task‐Monitor*), but not for *Emotional Control* or *Organisation of Materials*.

Here again, the EF profiles were investigated by running two different repeated‐measures ANOVAs. In one, the three indexes were dependent variables, and *Index* was the within‐group variable. In the other, the nine scales were dependent variables, and *Scale* was the within‐group variable. *T*‐scores were considered for both analyses.

A significant effect of *Index* emerged, *F*_2,118_ = 13.40, *P* < 0.001, *η*_p_^2^ = 0.19, and subsequent post‐hoc analyses showed higher scores in the *Cognitive Regulation Index* than in the *Behaviour Regulation Index* (*Mdiff*. = 4.15, *P* < 0.001, *d* = 0.59) or *Emotion Regulation Index* (*Mdiff*. = 5.07, *P* < 0.001, *d* = 0.59).

**FIGURE 2:**
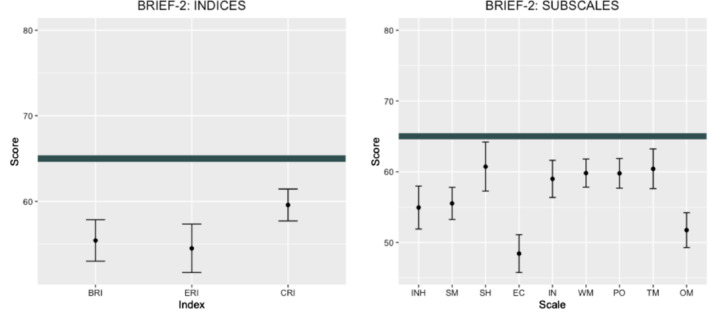
BRIEF‐2 scores in the school‐age group. BRI, Behaviour Regulation Index; CRI, Cognitive Regulation Index; BRIEF‐P, Behaviour Rating Inventory of Executive Function – Preschool Version; EC, Emotional Control; ERI, Emotion Regulation Index; IN, Initiate; INH, Inhibit; OM, Organisation of Materials; PO, Plan/Organise; SH, Shift; SM, Self‐Monitor; TM, Task‐Monitor; WM, Working Memory. The thick line represents the BRIEF‐P cut‐off of 65. Scores higher than this value are in the clinically elevated range. [Colour figure can be viewed at wileyonlinelibrary.com]

There was also a significant effect of *Scale*, *F*_6.39,376.98_ = 16.61, *P*_gg_ < 0.001, *η*_p_^2^ = 0.22. As shown in Fig. 2, *Emotional Control* and *Organisation of Materials* scored the lowest, and *Shift* the highest. Post‐hoc analyses identified significant differences between *Emotional Control* and *Inhibit* (*Mdiff*. = −6.52, *P* < 0.001, *d* = 0.72), *Self‐Monitor* (*Mdiff*. = −7.10, *P* = 0.003, *d* = 0.55), *Shift* (*Mdiff*. = −12.30, *P* < 0.001, *d* = 0.92), *Initiate* (*Mdiff*. = −10.57, *P* < 0.001, *d* = 0.86), *Working Memory* (*Mdiff*. = −11.38, *P* < 0.001, *d* = 1.15), *Plan/Organise* (*Mdiff*. = −11.35, *P* < 0.001, *d* = 1.02) and *Task‐Monitor* (*Mdiff*. = −11.98, *P* < 0.001, *d* = 0.97). *Organisation of Materials* differed significantly from *Initiate* (*Mdiff*. = −7.20, *P* < 0.001, *d* = 0.61), *Working Memory* (*Mdiff*. = −8.07, *P* < 0.001, *d* = 0.76), *Plan/Organise* (*Mdiff*. = −8.03, *P* < 0.001, *d* = 0.76), *Shift* (*Mdiff*. = −8.98, *P* < 0.001, *d* = 0.73) and *Task‐Monitor* (*Mdiff*. = −8.66, *P* < 0.001, *d* = 0.71). *Shift* differed from *Inhibit* (*Mdiff*. = 5.78, *P* = 0.04, *d* = 0.44).

#### Executive functions: profile comparison

**FIGURE 3:**
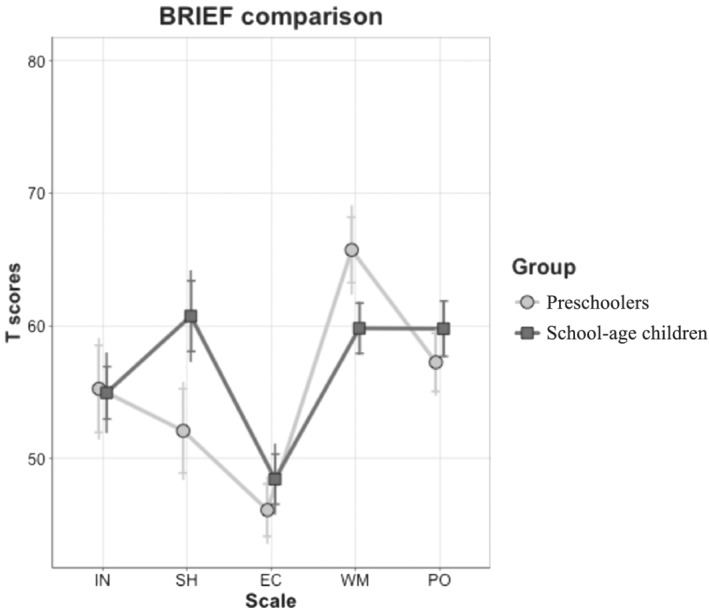
BRIEF profile comparison. BRIEF, Behaviour Rating Inventory of Executive Function; EC, Emotional Control; INH, Inhibit; PO, Plan/Organise; SH, Shift; WM, Working Memory.

Because the BRIEF‐P and BRIEF 2 have five scales in common (*Inhibit*, *Shift*, *Emotional Control*, *Working Memory* and *Plan/Organise*), the EF profiles of the two groups could be compared with a 5 × 2 ANOVA, with *Scale* as the within factor and *Group* as the between factor. The main effect of *Scale* (*F*_3.38,321.41_ = 45.34, *P*_gg_ < 0.001, *η*_p_^2^ = 0.31) and the *ScaleXGroup* interaction (*F*_3.38,321.41_ = 9.73, *P*_gg_ < 0.001, *η*_p_^2^ = 0.09) were significant, but the main effect of *Group* was not. Table 4 shows the corresponding post‐hoc analyses, and Fig. 3 is a graphical representation of the data.

**TABLE 4:** Post‐hoc analyses: BRIEF between groups and within group comparisons

		*Mdiff*	*P* value	Cohen's *d*
Between comparison	IN	0.30	1.00	0.01
	SH	−8.66	0.002	0.41
	EC	−2.33	1.00	0.11
	WM	5.91	0.25	0.28
	PO	−2.53	1.00	0.12
Within comparison	Preschoolers			
	IN vs. SH	3.18	1.00	0.17
	IN vs. EC	9.15	<0.001	0.50
	IN vs. WM	−10.48	<0.001	0.56
	IN vs. PO	−2.00	1.00	0.11
	SH vs. EC	5.97	0.06	0.32
	SH vs. WM	−13.75	<0.001	0.74
	SH vs. PO	−5.18	0.24	0.28
	EC vs. WM	−19.63	<0.001	1.06
	EC vs. PO	−11–15	<0.001	0.60
	WM vs. PO	8.47	<0.001	0.46
	School‐age children			
	IN vs. SH	−5.78	0.007	0.38
	IN vs. EC	6.52	<0.001	0.43
	IN vs. WM	−4.87	0.06	0.32
	IN vs. PO	−4.83	0.08	0.32
	SH vs. EC	12.30	<0.001	0.81
	SH vs. WM	−4.99	0.85	0.24
	SH vs. PO	0.95	1.00	0.06
	EC vs. WM	−11.38	<0.001	0.75
	EC vs. PO	−11.35	0.002	0.42
	WM vs. PO	0.03	1.00	0.002

BRIEF, Behaviour Rating Inventory of Executive Function; EC, Emotional Control; IN, Inhibit; PO, Plan/Organise; SH, Shift; WM, Working Memory.

Between‐group comparisons showed a significant difference in *Shift*, the older group having higher scores. Within‐group comparisons indicated that both groups had *Emotional Control* as a strength and *Working Memory* as a weakness, while it was only in the school‐age group that difficulties also emerged in *Shift* and *Plan/Organise*.

### Adaptive behaviour

#### Adaptive behaviour in the preschooler group

Table 5 shows the descriptive statistics, the percentages of clinically low standardised scores (<70) and the results of Student's *t*‐test comparing the mean *T*‐scores of the group with the norm of 100.

**TABLE 5:** Percentages of clinically high standard scores, means, standard deviations (SDs) and one‐sample *t*‐test results on the Vineland Adaptive Behaviour Scales, Second Edition

	%CE^†^	Mean	SD	*t* ^‡^	*P* value	Cohen's *d*
Preschoolers						
Communication	85	55.75	14.07	−19.88	<0.001	3.14
Daily Living Skills	70	62.28	13.00	−18.35	<0.001	2.90
Socialisation	22.5	74.58	13.23	−12.15	<0.001	1.92
Motor Skills	87.5	55.70	12.78	−21.93	<0.001	3.48
Adaptive Behaviour Composite score	82.5	55.73	11.96	−23.41	<0.001	3.70
School‐age children						
Communication	85	49.72	18.83	−20.69	<0.001	2.67
Daily Living Skills	86.7	50.22	18.82	−20.48	<0.001	2.65
Socialisation	70	60.60	17.45	−17.49	<0.001	2.26
Adaptive Behaviour Composite score	78.6	46.91	18.46	−27.26	<0.001	2.87

^†^ Percentage of individuals with DS reportedly in the low range (*T* < 70).

^‡^ Comparison with normative standardised score of 100.

Standardised scores obtained from the *Adaptive Behaviour Composite* scores were significantly below the norm of 100. Among the children with DS aged 3–6.11 years, far more than half of the sample had clinically low standardised scores on *Communication*, *Daily Living Skills* and *Motor Skills* (85%, 70% and 87.5%, respectively). In contrast, only 20% of the sample showed clinically low standardised scores for *Socialisation*.

#### Adaptive behaviour in the school‐age group

Table 5 also shows the descriptive statistics, percentages of clinically low standardised scores (<70) and results of Student's *t*‐test comparing the mean *T*‐scores of this group with the norm of 100. Standardised scores from the *Adaptive Behaviour Composite* scores were significantly below the norm of 100. Among these older children with DS, most of the sample had clinically low standardised scores in all three domains: *Communication, Daily Living Skills* and *Socialisation* (85%, 86.7% and 70%, respectively).

#### Adaptive behaviour: group comparison

**FIGURE 4:**
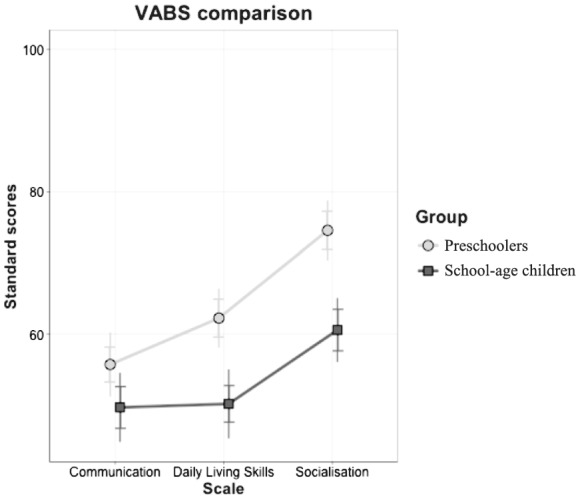
VABS profile comparison. VABS, Vineland Adaptive Behaviour Scales.

The two age groups were compared using a repeated‐measures ANOVA, where *Scale* was the within factor and *Group* the between factor (refer to Fig. 4 for a graphical representation). The main effects of *Scale* (*F*_2,196_ = 59.00, *P* < 0.001, *η*_p_^2^ = 0.37) and *Group* were significant (*F*_1,98_ = 13.06, *P* < 0.001, *η*_p_^2^ = 0.12), and so was the *ScaleXGroup* interaction (*F*_2,196_ = 4.20, *P* = 0.016, *η*_p_^2^ = 0.04).

Post‐hoc analyses on the effect of *Scale* showed that *Socialisation* was the highest score, and it differed significantly from *Communication* (*Mdiff*. = 14.06, *P* < 0.001, *d* = 0.93) and *Daily Living Skills* (*Mdiff*. = 11.15, *P* < 0.001, *d* = 0.81). Table 6 shows the post‐hoc analyses run after the significant interaction. Based on Bonferroni's correction, the alpha levels were adjusted to 0.016 (i.e. 0.05/3) for the comparisons between the groups on each scale, and to 0.008 (i.e. 0.05/6) for comparisons between the scales within each group.

**TABLE 6:** Post‐hoc analyses: Vineland Adaptive Behaviour Scales between groups and within group comparisons

		*Mdiff*	*P* value	Cohen's *d*
Between comparison	Communication	6.30	1.00	0.17
	Daily Living Skills	12.06	0.007	0.36
	Socialisation	13.98	<0.001	0.41
Within comparison	Preschoolers			
	Communication vs. Daily Living Skills	−6.53	0.05	0.29
	Communication vs. Socialisation	−18.83	<0.001	0.85
	Daily Living Skills vs. Socialisation	−12.30	<0.001	0.55
	School‐age children			
	Communication vs. Daily Living Skills	−0.50	1.00	0.03
	Communication vs. Socialisation	−10.88	<0.001	0.60
	Daily Living Skills vs. Socialisation	−10.38	<0.001	0.57

Between‐group comparisons indicated significant differences in *Daily Living Skills* and *Socialisation*, with the younger group obtaining higher standardised scores. Within‐group comparisons showed a similar picture for the two groups, with *Communication* and *Daily Living Skills* differing significantly from *Socialisation*, which emerged as a relative strength.

### Executive functions and adaptive behaviour

#### Correlations between VABS‐II and BRIEF‐P/BRIEF 2

The relationship between the VABS‐II scales and the BRIEF‐P and BRIEF 2 indexes was investigated, and the bivariate correlations between the VABS‐II standard scores and the BRIEF T‐scores in the two age groups are reported (Table 7 for BRIEF‐P, Table 8 for BRIEF 2).

**TABLE 7:** Correlations between BRIEF‐P and VABS in the preschooler group

	Communication	Daily Living Skills	Socialisation
Index			
Inhibitory Self‐Control Index^†^	−0.22	0.04	−0.08
Flexibility Index^‡^	−0.17	−0.01	−0.13
Emergent Metacognition Index^§^	−0.33*	−0.23	−0.20
Subscale			
Inhibit	−0.24	0.04	−0.09
Shift	−0.15	−0.04	−0.12
Emotional Control	−0.17	0.10	−0.13
Working Memory	−0.35†*	−0.15	−0.26
Plan/Organise	−0.21	−0.11	−0.14

BRIEF‐P, Behaviour Rating Inventory of Executive Function – Preschool Version; VABS, Vineland Adaptive Behaviour Scales.

^†^ Inhibitory Self‐Control = Inhibit + Emotional Control.

^‡^ Flexibility = Shift + Emotional Control.

^§^ Emergent Metacognition = Working Memory + Plan/Organise.

**TABLE 8:** Correlations between BRIEF 2 and VABS in the school‐age group

	Communication	Daily Living Skills	Socialisation
Index			
Behaviour Regulation Index^†^	−0.48***	−0.47***	−0.49***
Emotion Regulation Index^‡^	−0.41***	−0.46***	−0.50***
Cognitive Regulation Index^§^	−0.50***	−0.43***	−0.45**
Subscale			
Inhibit	−0.41***	−0.45***	−0.41***
Self‐Monitor	−0.31*	−0.20	−0.27*
Shift	−0.34**	−0.38**	−0.47***
Emotional Control	−0.33**	−0.37**	−0.34**
Initiate	−0.39**	−0.38**	−0.31**
Working Memory	−0.55***	−0.39**	−0.33**
Plan/Organise	−0.27*	−0.29*	−0.31**
Task‐Monitor	−0.34**	−0.19	−0.33**
Organisation of Materials	−0.28*	−0.36**	−0.36**

BRIEF, Behaviour Rating Inventory of Executive Function; VABS, Vineland Adaptive Behaviour Scales.

^†^ Behaviour Regulation = Inhibit + Self‐Monitor.

^‡^ Emotion Regulation = Shift + Emotional Control.

^§^ Cognitive Regulation = Initiate + Working Memory + Plan/Organise + Organisation of Materials + Task‐Monitor.

In the younger group, the only significant (moderate) correlation that came to light was between the *Emergent Metacognition Index* and *Communication*. This correlation is explained mainly by the significant correlation between *Working Memory* and *Communication*. In the older group, on the other hand, all three indexes (*Behaviour Regulation, Emotion Regulation* and *Cognitive Regulation*), and almost all the scales correlated significantly with the three adaptive behaviour indexes (*Communication, Daily Living Skills* and *Socialisation*).

#### Regression analyses

Simple linear regressions were run for both groups to examine the role of EFs (BRIEF‐P and BRIEF 2) on the children's adaptive behaviour (Vineland‐II). For each model, age was entered first, followed by the indexes (*ISCI*, *FI* and *EMI* for BRIEF‐P, and *BRI*, *ERI* and *CRI* for BRIEF 2), while each Vineland‐II scale (*Communication*, *Daily Living Skills* and *Socialisation*) was the outcome variable. When an index emerged as a predictor, further analyses were run to detect which scale of the index was most predictive.

For the younger group, of all the EF indexes and adaptive behaviour scores, the *Emergent Metacognition Index* was the only significant predictor (*β* = −0.33, *P* = 0.04) for *Communication*. Within the subscales comprising this index (*Working Memory* and *Plan/Organise*), *Working Memory* emerged as the significant predictor (*β* = −0.34, *P* = 0.03). No significant predictors were identified for *Socialisation* or *Daily Living Skills*.

For the older group, the analyses showed that the *Cognitive Regulation Index* was a predictor of *Communication* (*β* = −0.33, *P* = 0.04). In subsequent analyses on the scales comprising this index, *Working Memory* emerged as the significant predictor (*β* = −0.55, *P* < 0.001). The *Behaviour Regulation Index* and age were predictors of *Daily Living Skills* (*β* = −0.46, *P* < 0.001, *β* = −0.26, *P* = 0.03 respectively), and the *Inhibit* scale was the significant predictor (*β* = −0.45, *P* < 0.001). Two indexes, *Behaviour Regulation* and *Emotion Regulation*, together with age, were predictive of *Socialisation* (*β* = −0.31, *P* = 0.01, *β* = −0.27, *P* = 0.03, *β* = −0.49, *P* < .001 respectively). Then, when the single scales were considered, *Inhibit* was identified as the significant predictor (*β* = −0.41, *P* = 0.001), together with *Shift* (*β* = −0.47, *P* < 0.001).

## Discussion

The aim of this study was to clarify the EF and adaptive behaviour profiles of children/adolescents with DS, explore any differences by age, and analyse the relationship between EFs and adaptive behaviour in this particular population. Two groups of individuals with DS, one aged 3 to 6.11, the other aged 7 to 16 years, were assessed on their EFs (with the BRIEF 2/BRIEF‐P) and adaptive behaviour (with the VABS‐II).

### Executive functions

Generalised EF difficulties were seen in individuals with DS, consistently with the previous literature (e.g. Lee *et al*. 2011; Loveall *et al*. 2017). As in other studies (Daunhauer, Fidler, Hahn *et al*. 2014), there was no difference between the two age groups in terms of the severity of these difficulties described by parents. The preschooler group showed a relative strength in *Emotional Control*, while they were more impaired in *Shift*, *Plan/Organise* and *Inhibit*, and most impaired in *Working Memory*. The school‐age group showed a relative strength in *Emotional Control* and *Organisation of Materials*; intermediate ability levels for *Inhibit* and *Self‐Monitor*; and a relative weakness in *Shift*, *Initiate*, *Working Memory*, *Plan/Organise*, and *Task‐Monitor*. There were some similarities and some differences between the two age groups when the scales common to the two versions of the BRIEF were compared. Both groups were relatively strong on *Emotional Control* and weak on *Working Memory*, with *Inhibit* in between. The two age groups differed as regards *Plan/Organise* and *Shift*, domains in which the older children showed a more severe weakness. The greater difficulty in *Shift* and *Plan/Organise* in the older group may be because tasks in these domains get harder with age, or because the increasing demands at school make these difficulties more obvious to parents while they might previously have gone unnoticed (Loveall *et al*. 2017). Overall, these findings confirm the profile described in the literature (Daunhauer, Fidler, Hahn *et al*. 2014; Lee *et al*. 2011, 2015; Loveall *et al*. 2017), suggesting that some EFs remain stable over time, while others vary. It is worth noting that the results of the present study largely replicate those reported by Loveall *et al*. (2017), although the two studies examined slightly different age ranges due to the two countries where the studies were conducted adopting different policies on the age at which children start school. This seems to support the hypothesis that the environment, rather than age itself, might shape the EF profile of children with DS. More studies are needed to clarify this picture. Cross‐cultural studies on children aged between 5 and 7 years would be especially helpful in elucidating this matter.

### Adaptive behaviour

All the children with DS in the sample had standardised scores that were two standard deviations below the norm in at least one adaptive behaviour domain, and most of the children had deficits in more than one. This is in line with previous reports (e.g. Dykens *et al*. 2006; Will *et al*. 2018) and consistent with DS involving intellectual disability. Comparing the two age groups, preschoolers had higher standardised scores than school‐age children with DS in *Daily Living Skills* and *Socialisation*. The lower scores seen in the older children reflect not a loss of their abilities, but a slower development of these skills than in the typically developing population. Scores for *Communication* were equally low in both DS groups, in line with the tendency of individuals with this syndrome to be particularly weak in language development at any age (Silverman 2007; Grieco *et al*. 2015).

Similarities and differences also emerged in the two groups' adaptive behaviour. They were both relatively strong on *Socialisation* and weak on *Communication* and *Daily Living Skills*, but preschoolers scored higher for *Daily Living Skills* than school‐age children. These results are in line with previous reports of a relative strength in *Socialisation* and weakness in *Communication* (Dykens *et al*. 2006; Fidler *et al*. 2006; Van Duijn *et al*. 2010; Will *et al*. 2018; Spiridigliozzi *et al*. 2019) in children with DS. More variability has been reported regarding *Daily Living Skills*, and it may be that environmental variables (such as living conditions or education) have a role in modulating the development of these skills.

### Executive functions and adaptive behaviour

The relationship between EFs and adaptive behaviour differed in the two age groups considered here. The two domains seemed quite independent in preschoolers, with significant relationships only between *Communication* and *Working Memory* (the latter predicting the former). For school‐age children, on the other hand, correlations emerged between almost all EFs and adaptive behaviour domains, emphasising the important role of EFs in everyday functioning at this age. The *Emergent Metacognition Index* predicted *Communication*, with *Working Memory* emerging as the significant predictor. This can be explained by the relationship between working memory and language, which has been demonstrated in both typical development (Gathercole 2006) and DS (Lanfranchi *et al*. 2009). The *Behaviour Regulation Index*, and *Inhibit* in particular (an area where individuals with DS are weak; e.g. Lanfranchi *et al*. 2010; Borella *et al*. 2013), predicted *Daily Living Skills*. In daily living activities, such as self‐care, household tasks, managing money, time and technology, and coping with rules, it is important to inhibit a prepotent response in favour of a more pondered behaviour. The deficit in inhibition exhibited by individuals with DS may therefore play a part in their weak daily living skills. Moreover, *Inhibit* and *Shift* were the predictors of *Socialisation*. Looking at the subscales of the VABS in the socialisation domain (*Interpersonal Relationships, Play and Leisure, and Coping Skills*), inhibition – in the sense of our ability to monitor our behaviour, stop any inappropriate behaviour and choose a more appropriate response – plays an important part in relations with others, when playing or interacting in other ways. Porter *et al*. (2007) reported similar findings and associated this result with frontal lobe abnormalities. Shifting – or the ability to switch spontaneously to a different action, thought or person in response to situational changes – is also essential when interacting with others. It is used when playing games, for instance, to switch from one activity to another, and when social rules have to be applied to interpret a context (which can change rapidly) and decide what behaviour to adopt. Our results are consistent with findings in typically developing samples (e.g. Benavides‐Nieto *et al*. 2017; Kaushanskaya *et al*. 2017; Fogel *et al*. 2020), and in adolescents with DS (Sabat *et al*. 2020), suggesting that EFs support adaptive behaviour in DS in much the same way as in typical development.

Finally, it is worth noting the different involvement of EFs in adaptive behaviour at different ages. The preschoolers' adaptive behaviour demanded basic skills, such as understanding simple, common words, eating finger food or showing interest in others, that do not seem to be particularly supported by EFs. The older group's adaptive behaviour needed to be more complex and articulated, and this probably meant a greater involvement of their EFs.

### Limitations

Some limitations of this study need to be acknowledged. First, the data reported are cross‐sectional, not longitudinal, and this prevents us from giving a more detailed account of how EFs, adaptive behaviour and their interaction change over time. Comparing two cohorts might also have led to the possibility of other variables, besides age, contributing to group differences. Future work should track samples longitudinally to obtain a more precise picture of their developmental trajectories. Second, the present study relied on indirect measures, and parents may have different expectations depending on their children's age and their own mental representations of them – aspects that may have influenced the information they provided. It would be helpful to replicate the findings from this study using direct measures to track the developmental trajectories of EFs in individuals with DS.

### Implications

Understanding the relative strengths and weaknesses in the EFs and adaptive behaviour of individuals with DS, how they are related, and how they change over time could help us to pinpoint areas where extra support may be needed. Our results support the conviction that interventions targeting EFs are particularly important for individuals with DS, not only for their cognitive development but also to support their adaptive behaviour. For instance, knowing that EFs and adaptive behaviour are associated in school‐age children suggests that it might be important to target EFs in early intervention to support the abilities that are associated with adaptive behaviour in older children. Moreover, considering the association between EFs and adaptive behaviour, it would be interesting to envisage an intervention programme that involves exercises embedded in everyday life activities to foster EFs.

## Conflicts of Interest

No conflicts of interest have been declared.

## Source of Funding

The fellowship for G.R. has been funded by various overwhelmingly successful fundraising events in London organized by Alberto Ospite and Diego Discepoli, as well as to Laura Buonfino, Stefano Marzeglia, and Marco Pasinetti as documented at the following sites: https://www.justgiving.com/crowdfunding/genome21 or /genome21research. The fellowship for F.A. has been funded by donations from the Fondazione Umano Progresso and Matteo and Elisa Mele. The fellowship for B.V. has been funded by donations from ‘Associazione Amicorum’ and ‘Associazione più di 21 onlus’, Cassano Magnago (VA).

## Data Availability

The data that support the findings of this study are available from the corresponding authors upon reasonable request.
